# Initiation of adaptive feeding within 24 h after burn injury improves nutritional therapy for severely burned patients

**DOI:** 10.3389/fnut.2024.1342682

**Published:** 2024-06-26

**Authors:** Yin Zhang, Yi Dou, Zongqi Yin, Min Gao, Beiwen Wu, Qin Zhang

**Affiliations:** ^1^Department of Burn, Ruijin Hospital, Shanghai Jiao Tong University School of Medicine, Shanghai, China; ^2^Department of Nursing, Ruijin Hospital, Shanghai Jiao Tong University School of Medicine, Shanghai, China

**Keywords:** fasting, adaptive feeding, nutritional treatment, gastrointestinal dysfunction, severe burn

## Abstract

**Introduction and objective:**

Nutritional therapy is a crucial component of treatment for severely burned patients. Although overly aggressive enteral nutrition immediately after burn injury has potential risks, nutritional disruption after a severe burn can also increase infection risk and delay wound healing. For approximately six decades, the Ruijin Hospital Burn Center has used two distinct approaches for patients during the early period after burn injury: complete fasting or adaptive feeding. Notably, adaptive feeding more closely resembles enteral nutrition. In this retrospective study, we analyzed factors influencing the selection of either modality, as well as the benefits of adaptive feeding. We sought to promote adaptive feeding as a precursor to initiating enteral nutrition.

**Patients and methods:**

This retrospective study analyzed medical data from adult patients with extensive burns between January 2009 and December 2020. All patients had been admitted to the burn department within 24 h after injury and had a burned area comprising >30% of total body surface area. Patients were divided into two groups: adaptive feeding and fasting. We examined the total burned area, full-thickness burned area, burn type, inhalation injury, start time of adaptive feeding, and start time of enteral nutrition. Outcome measures were 28-day mortality and hospital mortality.

**Results:**

Univariate analysis revealed significant differences in burn type, percent of total body surface area (TBSA), full-thickness burned area, and inhalation injury between the adaptive feeding and fasting groups (all *p* < 0.05). Linear regression analysis showed that full-thickness burned area and inhalation injury were the main factors influencing the start time of adaptive feeding in patients with severe burns (*p* < 0.05). After propensity score matching analysis, the results showed that the start time of enteral nutrition was significantly earlier in the adaptive feeding group (*p* < 0.01). However, overall mortality, 28-day mortality, and length of hospital stay did not significantly improve in the adaptive feeding group. The incidence of intolerance after enteral nutrition therapy did not significantly differ between groups.

**Conclusion:**

The results of the study showed that larger full-thickness burned areas and concomitant inhalation injury were the primary factors considered by physicians when selecting complete fasting for severely burned patients. Moreover, the results indicate that adaptive feeding improves nutritional therapy for severely burned patients by shortening the time between injury and initiation of enteral nutrition. Complete fasting due to concerns about extensive burned area and inhalation injuries does not reduce the incidence of enteral nutrition intolerance; instead, it delays the initiation of enteral nutrition.

## 1 Introduction

Nutritional therapy is a crucial component of treatment for severely burned patients (1). In clinical practice, we have observed risks associated with the initiation of overly aggressive enteral nutritional support immediately after burn injury (2). Patients with severe burns may experience acute colonic pseudo-obstruction (3). Additionally, severe burn-induced edema within the gastrointestinal mucosa can cause nutrient malabsorption (4). There is a clinical consensus that gastrointestinal and metabolic processes can be disrupted by excessive or inappropriate nutritional therapy approaches (5). However, nutritional disruption after severe burn injury can also increase infection risk and delay wound healing. Therefore, the optimization of enteral nutrition timing is a key goal of clinical treatment.

For more than six decades, the Burn Center of Ruijin Hospital, the largest burn center in Shanghai, has not routinely administered enteral nutrition within 24 h of severe burn injury. Instead, two approaches have been used: complete fasting or adaptive feeding (e.g., approximately 300 mL of 5% glucose solution during the first 24 h) during the early period after burn injury. Notably, adaptive feeding more closely resembles enteral nutrition. A physician’s selection of complete fasting or adaptive feeding prior to enteral nutrition is based on their own clinical experience and (usually) patient characteristics such as burn area and inhalation injury. In this retrospective study, we analyzed factors influencing the selection of either modality, as well as the benefits of adaptive feeding, based on medical data from severely burned patients over the past 11 years. We sought to promote adaptive feeding as a precursor to initiating enteral nutrition.

## 2 Materials and methods

### 2.1 Study population

This retrospective study analyzed the medical records of severely burned patients who were admitted to Shanghai Ruijin Hospital between January 2009 and December 2020. According to the burn severity classification of the Chinese Burn Association, the patients in this study had a burn area comprising >30% of total body surface area (TBSA). Patients aged ≥18 years, admitted directly to the burn department within 24 h after burn injury, and with a total burned area comprising >30% of TBSA were included in the study. Patients with a history of medical treatment (e.g., anabolic hormones, immunosuppressants, or anti-tumor drugs) for an extended period before the burn injury and patients with a history of drug addiction were excluded from the study (Table 1).

**Table 1:** Independent variable assignment.

Variables	Assignment method
Burn type	Set dummy variables with fire as reference, dummy variable X1: fire = 1, scald, chemical and electric = 0. Dummy variable X2: scald =1, fire, chemical and electric =0. Dummy variable X3: chemical =1, fire, scald and electric burn = 0. Dummy variable X4: electric =1, fire, scald and chemic =0.
TBSA	30–50% TBSA = 1, greater than 50% TBSA = 0.
Full-thickness burned area	Less than or equal to 20% TBSA = 1, more than 20% TBSA = 0.
Inhalation injury	With inhalation injury = 1, without inhalation injury = 0.

### 2.2 Data collection

In this study, a standardized form was used to collect general data (age, sex, body mass index, time between injury and admission, and length of hospital stay) and clinical data (total burned area, full-thickness burned area, burn type, inhalation injury, start time of adaptive feeding, start time of enteral nutrition, and enteral nutrition-related gastrointestinal adverse reactions). Patients were divided into two groups, adaptive feeding and fasting, based on whether they began adaptive feeding within the first 24 h after injury. Outcome measures were 28-day mortality, hospital mortality, length of stay, incidence of gastrointestinal complications, and start time of enteral feeding.

All investigators underwent systematic training before collecting data from medical records, and all data were cross-checked for accuracy. This study was performed in accordance with the Declaration of Helsinki, and the study protocol was approved by the Research Ethics Committee of Ruijin Hospital, Shanghai Jiao Tong University School of Medicine (Decision no. 2022019).

### 2.3 Statistical analysis

Skewed data are presented as medians (interquartile ranges) and were compared using the Mann–Whitney test or Kruskal–Wallis test. Categorical data are presented as n (%) and were compared using the chi-square test. Kaplan–Meier survival analysis was utilized to assess the effects of adaptive feeding within 24 h on the 28-day survival rate. Multivariate linear regression analysis was performed to identify factors influencing the start time of adaptive feeding. Categorical variables were not directly entered into the regression equation because the suspected effect was assumed to be non-linear among categories; thus, dummy variables were used (*α*-values for selection were ≤ 0.5, whereas *α*-values for elimination were ≥ 1.00). Propensity score matching (PSM) was conducted to eliminate or control potential confounding factors caused by imbalances in baseline clinical data between the adaptive feeding and fasting groups. Matching variables were total burned area, full-thickness burned area, and inhalation injury; a caliper value of 0.02 was implemented. SPSS25.0 software was used for statistical analysis, and *p*-values <0.05 were considered statistically significant.

## 3 Results

In total, 601 patients with a total burn area comprising >30% of TBSA were eligible for inclusion in the study; 522 patients were included in the analysis. Seventy-nine patients were excluded because they had been transferred from other provinces to our burn center more than 24 h after injury. The excluded patients consisted of 58 men and 21 women, with a mean age of 48.11 ± 14.31 years and a mean transfer time of 55.37 ± 15.45 h after burn injury.

### 3.1 Univariate and multivariate analyses for factors associated with the start time of adaptive feeding

Univariate analysis showed that burn type, percent of TBSA, full-thickness burned area, and inhalation injury were significantly associated with the start time of adaptive feeding (all *p* < 0.05) (Table 2).

**Table 2:** Analysis of influencing factors for start time of adaptive feeding.

Variable	Group	*n*	Start time of adaptive feeding	*Z*/*X*^2^	*p* value
Age (years)	≤60	438	2.00 (0.00, 3.00)	0.528	0.597
	>60	84	2.00 (0.00, 3.00)		
Gender	Male	405	2.00 (0.00, 3.00)	−0.495	0.621
	Female	117	2.00 (0.00, 3.00)		
BMI (kg/m^2^)	<18.5	24	2.00 (1.00, 2.75)	1.719	0.633
	18.5–23.9	276	2.00 (0.00, 3.00)		
	24–27.9	176	2.00 (0.00, 3.00)		
	>28	46	2.00 (0.00, 3.00)		
Burn type	Fire	403	2.00 (0.00, 3.00)	8.354	0.039
	Scald	62	2.00 (0.00, 3.00)		
	Chemical	39	2.00 (0.00, 3.00)		
	Electric	18	2.00 (0.00, 3.00)		
TBSA	30–50%	243	2.00 (0.00, 3.00)	−4.220	0.000
	≥50%	279	2.00 (1.00, 3.00)		
Full-thickness burned area	≤20%	260	2.00 (0.00, 3.00)	−5.601	0.000
	>20%	262	2.00 (1.00, 3.00)		
Inhalation injury	Yes	200	3.00 (1.25, 4.00)	−5.901	0.000
	No	322	2.00 (1.00, 3.00)		

Multivariate linear stepwise regression was conducted with burn type, total burned area, full-thickness burned area, inhalation injury, and mechanical ventilation as independent variables. The results indicated that full-thickness burned area and inhalation injury were the main factors influencing the start time of adaptive feeding among severely burned patients (*p* < 0.05) (Table 3).

**Table 3:** Multivariate linear regression of influencing factors for the start time of adaptive feeding in severely burned patients.

Variable	*B*	Std error	Beta	*t*	*p* value
Constant	0.998	0.289		3.447	0.001
Inhalation injury	0.619	0.202	0.141	3.060	0.002
Full-thickness burned area	0.453	0.197	0.106	2.299	0.022

### 3.2 Survival analysis

**Figure 1:**
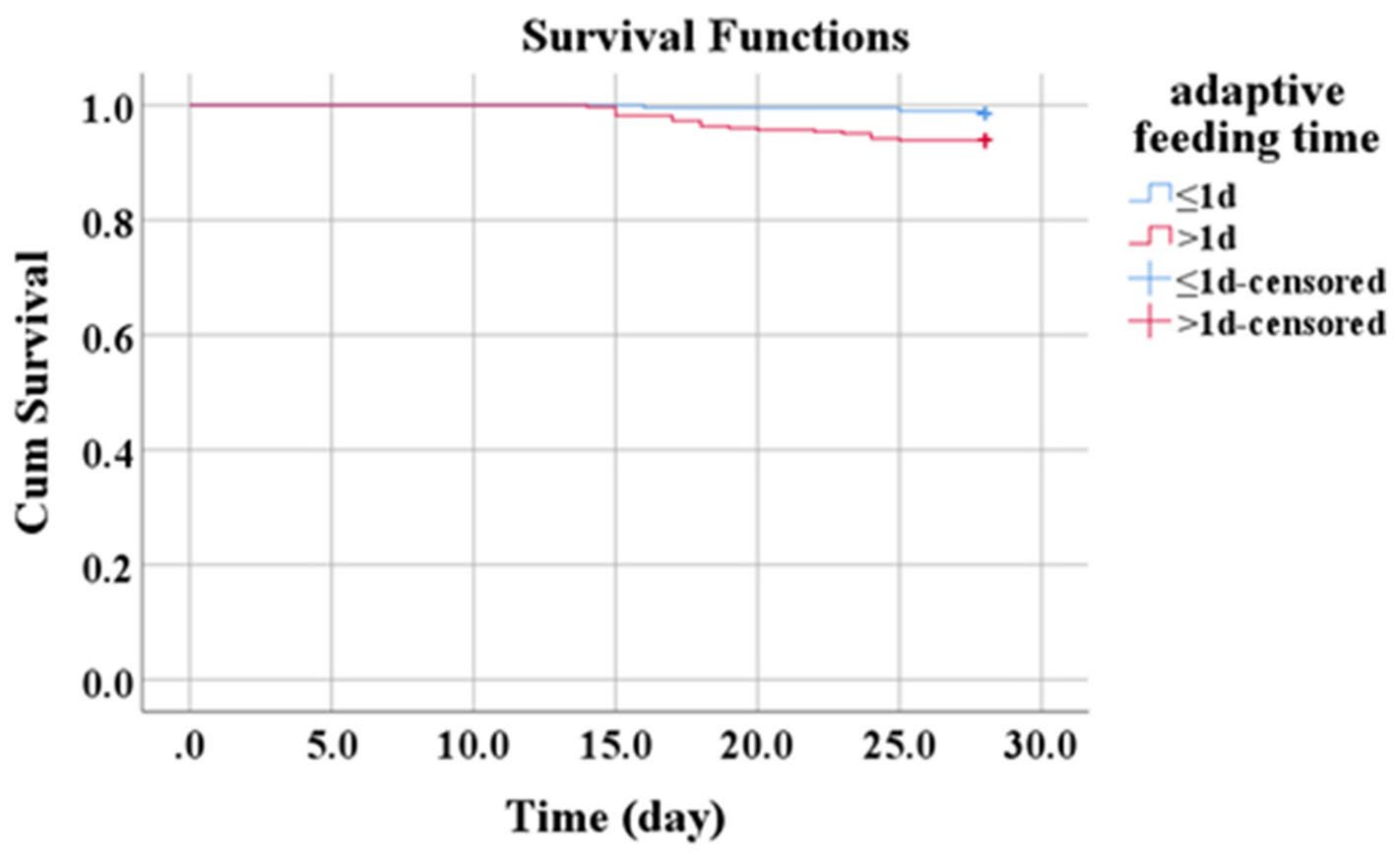
Survival analysis of the adaptive feeding group versus the fasting group.

Kaplan–Meier survival analysis was performed to examine whether the initiation of adaptive feeding within the first 24 h after burn injury influenced 28-day survival among patients with severe burns. In the adaptive feeding group, 3/195 patients died within 28 days (survival rate: 98.5%); in the fasting group, 20/327 patients died within 28 days (survival rate: 93.9%). Thus, patients who began adaptive feeding within 24 h after burn injury had a lower 28-day mortality rate (*X*^2^ = 6.083, *p* = 0.014, Figure 1).

### 3.3 Propensity score matching

The length of hospital stay was significantly shorter in the adaptive feeding group than in the fasting group (*p* < 0.01). Furthermore, patients in the adaptive feeding group began enteral nutrition earlier than those in the fasting group (*p* < 0.01). There was no significant difference in the incidence of enteral nutrition intolerance between the two groups of patients.

After PSM, there were no significant differences in overall mortality, 28-day mortality, length of hospital stay, or enteral nutrition intolerance between the two groups; however, the start time of enteral nutrition was significantly earlier in the adaptive feeding group (*p* < 0.01) (Table 4).

**Table 4:** Covariate balance between adaptive feeding group and fasting group.

Covariate	Before matching				After matching			
	Adaptive feeding within the first 24 h (*n* = 195)	Fasting within the first 24 h (*n* = 327)	*X*^2^/*Z*	*p* value	Adaptive feeding within the first 24 h (*n* = 181)	Fasting within the first 24 h (*n* = 181)	*X*^2^/*Z*	*p* value
Overall survival	190 (97.44%)	294 (89.91%)	10.255	0.001	176 (97.24%)	169 (93.37%)	3.024	0.082
28-day survival	192 (98.46%)	307 (93.88%)	6.078	0.014	178 (98.34%)	175 (96.69%)	1.025	0.502
Length of hospital stay	34.00 (24.00, 37.00)	38.00 (28.00, 57.25)	−2.763	0.006	35.00 (26.00, 48.75)	33.00 (25.50, 46.50)	0.543	0.587
Start time for enteral nutrition	3.00 (2.00, 4.00)	4.00 (3.00, 5.00)	−10.693	0.000	3.00 (2.00, 4.00)	4.00 (3.00, 5.00)	−7.973	0.000
Enteral nutrition intolerance	15 (7.69%)	34 (10.40%)	1.051	0.305	13 (7.18%)	15 (8.29%)	0.155	0.694

## 4 Discussion

To determine the optimal feeding strategy for critically burned patients before initiating enteral nutrition, this retrospective study explored the use of adaptive feeding immediately after burn injury as an appreciated approach. The results of the study showed that larger full-thickness burned areas and concomitant inhalation injury were the primary factors considered by physicians when selecting complete fasting for severely burned patients. Moreover, the results indicate that adaptive feeding improves nutritional therapy for severely burned patients by shortening the time between injury and initiation of enteral nutrition. Complete fasting due to concerns about extensive burned area and inhalation injuries does not reduce the incidence of enteral nutrition intolerance; instead, it delays the initiation of enteral nutrition.

These findings are consistent with previous research showing that extensive full-thickness burned areas and inhalation injury, both indicators of burn severity, influenced physicians’ decisions regarding complete fasting. Most physicians are hesitant to initiate aggressive enteral nutrition in critically ill patients. In 2017, Reintam Blaser et al. analyzed potential barriers to early enteral nutrition in 23 critical care scenarios. They found that acute lung injury and unstable hemodynamics during fluid resuscitation were concerns among physicians involved in administering enteral nutrition (6). There is disagreement regarding the use of enteral nutrition among critically ill patients who exhibit unstable hemodynamics during fluid resuscitation (7). Furthermore, McClave et al. reported that approximately one-third of critically ill patients in the intensive care unit (ICU) developed gastrointestinal motility disorders (8), whereas Sierp et al. reported that 51% of critically burned patients in the ICU developed gastrointestinal motility disorders (9). Extensively burned patients have a higher risk of gastrointestinal dyskinesia compared with non-burn patients admitted to the ICU (10). Patients with severe burns may experience acute colonic pseudo-obstruction and gastrointestinal mucosal edema, which are barriers to early enteral nutrition (11). Therefore, the use of enteral nutrition immediately after burn injury is not widely accepted (12). Clinical signs such as absent bowel sounds, abdominal/intestinal distention, gastrointestinal bleeding, and large gastric residual volumes are associated with mortality (13). It is important to determine the optimal approach before initiating enteral nutrition (14). Although adaptive feeding does not meet metabolic and energy needs, it avoids complete fasting in critically ill patients (15).

In this study, the implementation of adapting feeding immediately after burn injury did not reduce overall mortality, 28-day mortality, or length of hospital stay. These findings align with the results in many previous studies, which showed that larger full-thickness wound areas and inhalation injury hindered significant reductions of in-hospital mortality among severely burned patients (16). Fuentes Padilla et al. noted that it is unclear whether earlier enteral nutrition can decrease the risks of mortality, feeding intolerance, or gastrointestinal complications (17). Similarly, earlier normocaloric enteral nutrition did not reduce mortality (28-day and overall); duration of life support; lengths of stay in the ICU and hospital; and the rates of ICU, hospital, and 90-day mortality in adult shock patients receiving ventilatory support (18). Arabi et al. (19) suggested that early enteral feeding intolerance is an indicator of disease severity and a protective physiological response. Inappropriate enteral nutrition has been associated with poor ICU outcomes, particularly in the context of increased digestive complications (18). Safe and effective feeding in burn patients must be carefully balanced because acute colonic pseudo-obstruction and severe burn-induced edema within the gastrointestinal mucosa can cause nutrient malabsorption. To facilitate the recovery of gastrointestinal motility and metabolic processes in the initial phase after burn injury, lower calorie and protein feeding methods must become more widely accepted (20). Adaptive feeding is one strategy that reflects this approach.

There is no universally accepted approach to optimizing treatment before the implementation of enteral nutrition. Worldwide, patients do not receive nutrition for more than 60 h after ICU admission (21). Critically ill patients often cannot receive standard enteral nutrition as prescribed during the acute phase (22). In the present study, enteral nutrition was implemented significantly earlier in the adaptive feeding group, suggesting that adaptive feeding can reduce nutritional risk in severely burned patients compared with delayed initiation of enteral nutrition via complete fasting (23). This conclusion is consistent with the European Society for Clinical Nutrition and Metabolism (ESPEN) guideline regarding clinical nutrition in the ICU, which recommends initiating low-dose enteral nutrition within 24–48 h (11). However, early initiation of adaptive feeding does not reduce the incidence of enteral nutrition intolerance. This observation supports the hypothesis of Arabi et al. (19) that enteral nutrition intolerance is an indicator of disease severity and a systemic response. Furthermore, reliance on enteral nutrition or adaptive feeding does not reduce disease severity or modulate the systemic response (17). The main goal of adaptive feeding in severely burned patients is to safely initiate enteral nutrition soon after burn injury. From this perspective, adaptive feeding is an appropriate strategy (7). Complete fasting relies on passively waiting for bowel function to recover, potentially increasing the nutritional risk.

There are some limitations of our study. In particular, its single-center retrospective design may have led to incomplete results. In this study, some indicators of gastrointestinal motive function, such as bowel sounds, could not be quantified and analyzed due to the lack of complete data in medical records. In addition, gastric residual volume (GRV) measurement in patients with severely burned patients had not been possible for decades. Recently, ultrasound technology has been used to measure gastric residual volume. Such indicators reflecting the gastrointestinal motive function will be more detailed. Prospective multicenter studies involving measures of gastrointestinal motive function are needed to confirm the findings and address other limitations (24).

The results of the study showed that larger full-thickness burned areas and concomitant inhalation injury were the primary factors considered by physicians when selecting complete fasting for severely burned patients. Moreover, the results indicate that adaptive feeding improves nutritional therapy for severely burned patients by shortening the time between injury and initiation of enteral nutrition. Complete fasting due to concerns about extensive burned area and inhalation injuries does not reduce the incidence of enteral nutrition intolerance; instead, it delays the initiation of enteral nutrition.

## Data availability statement

The original contributions presented in the study are included in the article/supplementary material, further inquiries can be directed to the corresponding authors.

## Ethics statement

The data collecting and recording process was performed by the Declaration of Helsinki and the study was approved by the Research Ethics Committee of Ruijin Hospital, Shanghai Jiao Tong University School of Medicine (Decision no. 2022019). Written informed consent for participation in this study was provided by all participants.

## Author contributions

YZ: Data curation, Formal analysis, Methodology, Writing – original draft, Writing – review & editing. YD: Investigation, Methodology, Resources, Writing – review & editing. QZ: Conceptualization, Methodology, Writing – review & editing. ZY: Data curation, Investigation, Writing – review & editing. MG: Data curation, Formal analysis, Investigation, Writing – review & editing. BW: Conceptualization, Data curation, Investigation, Writing – review & editing.
